# A Spiking Neural Network Based Cortex-Like Mechanism and Application to Facial Expression Recognition

**DOI:** 10.1155/2012/946589

**Published:** 2012-10-30

**Authors:** Si-Yao Fu, Guo-Sheng Yang, Xin-Kai Kuai

**Affiliations:** School of Information and Engineering, The Central University of Nationalities, Beijing 100081, China

## Abstract

In this paper, we present a quantitative, highly structured cortex-simulated model, which can be simply described as feedforward, hierarchical simulation of ventral stream of visual cortex using biologically plausible, computationally convenient spiking neural network system. The motivation comes directly from recent pioneering works on detailed functional decomposition analysis of the feedforward pathway of the ventral stream of visual cortex and developments on artificial spiking neural networks (SNNs). By combining the logical structure of the cortical hierarchy and computing power of the spiking neuron model, a practical framework has been presented. As a proof of principle, we demonstrate our system on several facial expression recognition tasks. The proposed cortical-like feedforward hierarchy framework has the merit of capability of dealing with complicated pattern recognition problems, suggesting that, by combining the cognitive models with modern neurocomputational approaches, the neurosystematic approach to the study of cortex-like mechanism has the potential to extend our knowledge of brain mechanisms underlying the cognitive analysis and to advance theoretical models of how we recognize face or, more specifically, perceive other people's facial expression in a rich, dynamic, and complex environment, providing a new starting point for improved models of visual cortex-like mechanism.

## 1. Introduction

 Understanding how rapid exposure to visual stimuli (face, objects) affects categorical decision by cortical neuron networks is essential for understanding the relationship between implicit neural information encoding and explicit behavior analysis. Quantitative psychophysical and physiological experimental evidences support the theory that the visual information processing in cortex can be modeled as a hierarchy of increasingly sophisticated, sparsely coded representations, along the visual pathway [1], and that the encoding using pulses, as a basic means of information transfer, is optimal in terms of information transmission. Such a spiking hierarchy should have the unique ability of decorrelating the incoming visual signals, removing the redundant information, while preserving invariability, in an effort to maximize the information gain [2]. Therefore, characterizing and modeling the functions along the hierarchy, from early or intermediate stages such as lateral geniculate nucleus (LGN), or prime visual cortex (V1), are necessary steps for systematic studies for higher level, more comprehensive tasks such as object recognition. However, the detailed functional analysis still remain impractical, due to little quantitative work has been done to explore modeling the entire visual cortical system to selectively amplify important features to facilitate discrimination, neither viewed from computational intelligence point of view nor judged from neuroinformatics angle, making the idea of comprehensive analysis for cognition an attractive yet elusive goal.

Traditional approaches have tackled the issue by creating a computational network resembling the basic visual system in which information is processed through hierarchy model. Pioneering attempts include the Neocognitron by Fukushima [3], which processes information with rate-based neural units to deal with transformation invariant features, followed by the emergence of a bunch of functionally similar models, such as hierarchical machine proposed by LeCun and Bengio [4, 5], bottom-up model mechanism by Ullman et al. [6, 7], or model by Wersing and Körner [8]. This trend was later followed by a noticeable hierarchical cognitive model by Poggio in [9], using hierarchical layers similar to neocognition, and processing units based on softmax (MAX-like) operation. The combination makes the model applicable for the initial simulation of cortex-like mechanism. This biologically motivated hierarchical method was further carefully analyzed by Serre et al. on several real-world datasets in [10, 11], yielding comparable performance with benchmark algorithms. All together, a conclusion can be drawn that traditional views describe information coding in terms of components, such as frequency, intensity and orientation, which are estimated from the visual information. This perspective focuses more on the properties and response of the cortical neurosystems rather than its functional purpose. Therefore, although publications focused on this direction are large and lively and readers are referred to the following section to get a detailed survey for this trend, nevertheless, surprisingly little works attempted to explore the cognition mechanism using biologically inspired computing units.

In contrast, recent evidences from neurobiology have led researchers to build cortex-like scheme based model with single spiking neurons act as computation units (most cortical physiologists believe that most neurons in cortex spike), the so-called spiking neural networks (SNNs). Starting with the first successful spiking neuron model, Hodgkin-Huxley's model [12], several prototypes have subsequently been proposed, such as integrate-and-fire model (IF model) [13], one of the simplest yet most effective model describing the dynamic of the neurons, and its extension, spike response models (SRMs) [13], liquid state machine (LSM) [13], and Izhikevich model [14]. As a neurobiological plausible framework, SNNs have been confirmed showing great computational power, both theoretically and experimentally by several noteworthy works. Thorpe et al. proposed a three-layered, feedforward network (SpikeNet) using a fast IF neurons [15, 16]. Based on event-driven computation, SpikeNet have reported successfully tracking and recognizing faces in real time without increasing computation burden. Wysoski et al. [17, 18] introduced a spiking neural network framework with IF model and Hebbian learning rule, which is a hierarchical structure composed of four feedforward layer of neurons grouped in two neuronal maps. The whole system has been successfully testified with VidTimit dataset to recognize individuals using facial information. These solutions try to address the recognition problem by representing complex object into simple features concerned with orientation or spatial frequency, in order to ensure invariance with respect to scale and transformation. However, due to the correlated activity and sparse coding principle of the cortical neuron groups (often omitted in many models), the dynamics of the network can not be fully reflected by individual and isolated neurons. Understanding and incorporating the functional role of high-order correlations among neuron groups are necessary yet challenging task on which few works have been focused.

Motivated by the potential of SNN and hierarchy model, we address this issue in the context of the neural encoding and neural computing, here we propose a multilayer feedforward, hierarchical network consisting of integrate-and-fire neuron model that can successfully detect, analyze and recognize object of interest. Decoding orientation, locating position, reducing correlation and inferring category of object are conducted by subsequent, increasingly complex network level. As a proof of principle, we have implemented a prototype model and focused on testifying its performance on one unique category of objects—human facial expressions—a visually manifestation of human emotions—as a paradigm for understanding hierarchical processing in the ventral pathway. Neurobiological substrate of human emotion such as fear, anger, or disgust has long been an attractive goal because emotions are less encapsulated than other psychological states. Facial expression, in which human emotions are uniquely embodied and manifest, is one of the most direct ways that people coordinate, communicate emotions and other mental, social, and physiological cues. As the result, they are often considered as the shortcut to reveal the psychological consequences and mechanisms underlying the emotional modulation of cognition [19]. Therefore, the progress of research on facial expressions directly mirrors the wider advance in emotion analysis.

Generally speaking, applying such hierarchical structure to facial expression recognition is not a novel idea. Dailey et al. have already proposed a simple yet delicate prototype called EMPATH in [20] a decade ago, EMPATH is actually a biologically plausible neural network model for categorizing facial expressions. As a standard feedforward network which consists of three layers, EMPATH performs like Gabor filter in the first level, then extracted visual information representation is delivered to the second layer where dimension reduction is performed, and PCA is applied for image compression network, finally, the outputs of the decision making layer (gestalt layer) are categorized into the six basic emotions. The authors demonstrated the model's potential by using a simple facial expression dataset, the model has been applied for further analysis later in [21] with different facial expression datasets such as JAFFE, yielding satisfactory results. However, it should be noted that traditional linear analysis model (such as PCA) proposed for artificial, Gaussian like stimuli which can be fully described by second order correlations will suffer from the biased results as natural image (or faces captured outside under variant illumination or embedded in complex background, such as in the video security surveillance task) statistics tend to be highly nonGaussian, which may limit its further applications. Thus, techniques for capturing these higher order statistics to form efficient representations of visual information could be viewed as a natural solution.

Though we share a similar motivation as previous authors [16, 17], our approach is very different. Our paper makes two main contributions. First, we develop a novel framework that biologically mimic the operation scheme in visual pathway, which emphasizes the sparsity and efficiency of the visual cortex, specifically, the high order correlation is dealt by TICA. Second, we show how to apply the system to the practical pattern recognition tasks such as facial expression recognition. Several facial expression datasets are testified using the proposed approach, including frontal view, nonfrontal view, and illumination variant view. Though being fully aware that this attempt is a simplistic approximation of how the brain's real neural circuits truly operate, we still obtain satisfactory results.

The initial of the framework and some empirical experimental results have been appeared in the conference papers [22], here, we make the following modifications.

1. Carefully reexamine the whole framework and make a comprehensive, expliciting description.
2. Presenting several other experimental results.
3. Listing detailed discussions for the drawbacks and advantages of the model, and giving out the future possible improvement directions.

This rest of this paper is organized as follows.  Section 2 reviews the fundamental of visual system and current state of art of such hieratical cortex-like mechanism models.  Section 3 reviews the basics of spiking neuron models, followed by the proposal of our framework, the dynamics, structure, and the learning mechanism which are discussed in details. Several experimental results are shown in Section 5. We also provide some discussions and summaries, Section 6 concludes the final part. 

## 2. Visual System and Cortex Like Model: Current State of Art

 This section manly consists two large parts, we first begin our story by briefly reviewing the fundamentals of visual system, then we investigate the current correspondingly proposed methods and approaches. The pros and cons are discussed in detail. We particularly discussed the computing units, which would be used in the latter part. 

### 2.1. Vision System: Basics

 From retina to visual cortex, the neural circuits in our brain that underlie our cognitive behavior have evolved to be perfectly suited for processing visual information with remarkable efficiency, capable of prodigious computation, and marvels of communication [1]. Many existing approaches in computational neuroscience are based on the physiological observation that cognitive task are performed from simple to complex, through a hierarchical structure. The commonly accepted standard model of prime visual cortex briefly reviewed as follows.

1. Visual processing is a roughly feedforward, from low to high levels of the hierarchy. Early vision system creates representations at successive stages along the visual pathway, from retina to lateral geniculate nucleus (LGN) to V1, with a considerate data compression rate without noticeable information loss [23].
2. Neurons in V1 can be divided roughly into two classes, simple and complex, based on the spatial separation or overlap of their responses to light and dark stimuli, as well as bars and sinusoidal gratings. Simple cells have receptive fields (RFs) containing oriented subregions each responding exclusively to either light onset/dark offset (ON subregions) or dark onset/light offset (OFF subregions). Complex cells respond primarily to oriented edges and gratings, behaving like simple cells, however, they have a degree of spatial invariance [17].
3. Visual cortex is mainly consist of two routes [11, 12, 23, 24]: ventral stream and dorsal stream, the former is involved in the identification of objects and mostly found in the posterior/inferior part of the brain, while the latter is linked to the localization of objects and mostly found in the posterior/superior part of the brain.
4. From a neurocomputing perspective, neurons communicate with one another by sending encoded electrical impulses referred to as action potentials or spikes. Barlow [2] recognized the importance of information theory in this context and hypothesized that the efficient coding of visual information could serve as a fundamental constraint on neural processing. This hypothesis holds that a group of neurons should encode information as compactly as possible, so as to utilize the available computing resources most effectively.
5. The efficient coding hypothesis decouples naturally into two separate yet related statements. One regarding the statistics of individual neural responses and second regarding sparsity of the neural response. The responses of different neurons to the natural environment should be statistically independent from each other, thus, the information carried by each neuron should not be redundant with that carried by the others. This is also consistent with a notion that the visual system strives to decompose a scene into statistically independent constituents. Successful theoretical models include the independent component analysis (ICA) [25] and sparse coding [9, 26, 27].

### 2.2. Vision Hierarchy Model: State of the Arts

 What has those aforementioned theoretical components brought to the field of the emulation of brain-like process for the purpose of pattern recognition and categorical decision making? The consequences is the emerging of many models in which information is processed through several areas resembling the visual system. Pioneering biologically inspired attempts include the famous neocognitron, proposed by Fukushima and Miyake [3], which processes information with rate-based neural units, and LeCun et al. [4, 5], Ullman et al. [6, 7], Wesing and Koerner [8], all these models have been proven later to be qualitatively constrained by the anatomy and physiology of the visual cortex and may not actually suitable for practical computer vision systems. Thus, a more comprehensive, generic, high-level computational framework is required such that fast and accurate object recognition can be accomplished by summarizing and integrating huge amount of data from different levels of understanding, while keeping the trade-off between sparsity and discriminativeness, as well as gaining enough invariance for robust performance.

Recently, a cognitive model initialized by Riesenhuber et al. [9, 10], using hierarchical layers similar to neocognition, and processing units based on MAX-like operation, received sizeable concentration. The core of the model is the hypothesis that the main function of the ventral stream can be viewed as a mechanism which has evolved to achieve the trade-off between selectivity and invariance in IT area for fast and accurate object of interest recognition tasks, which is done through a underlying hierarchical structure (from retina to IT) with increasing invariance to object's appearances (rotation, scale, location, etc.)^1^. The model produces relative position and scale invariant features for object recognition.

The biologically motivated hierarchical method was further carefully analyzed by Serre et al. on several real-world datasets [10], by extracting shape and texture properties. The analysis encompassed invariance on single-object recognition and recognition of multiple objects in complex visual scenes (e.g. leaves, cars, faces, airplanes, motorcycles). The method presented comparable performance with benchmark algorithms. There have been a many great publications focused on this direction. For detailed survey paper we refer readers to Poggio and Serre's recent work on models of visual cortex [28]. 

### 2.3. Discussion

 Hierarchical representations began to dominate cognitive psychology and the following neuroscience in the 1960s. However, from the computational point of view, hierarchical model can be viewed as conceptual tools rather than computational means. Though sharing the inherent merit of being logically structured, being lack of computational units for the communication supports sometimes weaking the system's performance. How to combine the logic structure of the hierarchy with the computation unit in vivo should be considered with a great attention. This is what our paper aim for, thus, by incorporating artificial spiking neuron model (as computing unit) into the hieratical model, we come up with a novel cognitive framework which can be applied to some practice pattern recognition problems. The basic principle for spiking neural networks are presented in the following section. 

## 3. Spiking Neuron Model

 We first begin this section by briefly introducing the principle of SNN, which utilizes information representation as trains of spikes, embedded with spatiotemporal characteristics. Simplified integrate-and-fire neurons are deployed in the model, which discards the postsynaptic potential (PSP, stands for the activation level of the neuron) leakage, compared with the standard version. The main advantages of this neuron model is computationally inexpensive, and it boosts the importance of the first presynaptic spikes. the excitation depends on the order of arrival of spikes and the inactivation of neuron after the output spike (the PSP is permanently set to the resting potential level). The result is the implementation of a simplified general decoding scheme for input latencies [16, 17].

Every single neuron acts as a coincidence detection unit and the PSP for neuron *i* at a time *t* is calculated as

(1) $$\begin{array}{c} P\left(i,t\right)=\sum \text{mo}{\text{d}}^{\text{order}\;(j)}w_{j,i}, \end{array}$$

where mod⁡(*j*)∈(0,1) is the modulation factor, each time the neuron receives a spike, the efficiency of spike integration is divided by this factor, with the result that the earliest spikes have the strongest impact on the activation level (PSP). Thorpe demonstrated that the spatial-temporal structure of this first wave of spikes could carry nearly all the information in the input needed for further recognition, both rapidly and accurately [15, 29]. Order (*j*) is the firing rank of neuron, *w*_*j*,*i*_ represents the corresponding synaptic weight. According to [17], an output spike is generated if (and only if)

(2) $$\begin{array}{c} P\left(i,t\right)\geq P_{\text{th}}\left(i\right), \end{array}$$

where *P*_th_(*i*) is the postsynaptic threshold.

## 4. Network Topology

**Figure 1:**
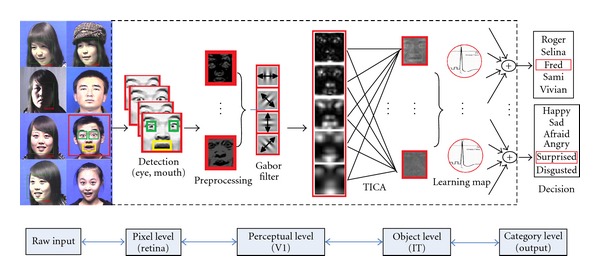
Framework of the entire facial expression recognition system. From the raw input to the final output, with each layer's illustration included, which have shown that the combination of selectiveness, determinativeness and invariance is built up gradually across several stages of facial information processing. The preprocessing part includes the detection of facial regions(eye, mouth, etc.), illumination normalization, retina level is also responsible for edge detector for enhancing the high contrast of the image. The second level functions like Gabor filter, which send the output to the perceptual level for extracting features which are robust for selectivity and invariance, then after being grouped and classified, the category level gives the output results (best view in color) [22].

**Figure 2:**
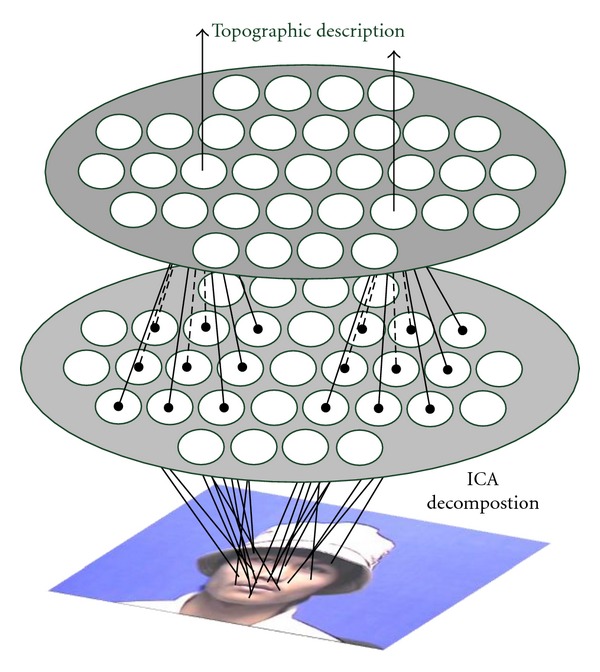
Two level scheme of TICA. The first level is the classic ICA extraction procedure, From the bottom to the top, the extracted components are gradually pooled into small neighbor, with increasing interaction among each other [22].

 Following the standard model of visual cortex [23], from the sensory/input layer to the final classification layer, the overall system consists of three main blocks: (1) the sensory/receptive layer, which consists of simple cell behavior simulator and complex cell behavior simulator, notice that sensory input and data preprocessing, including feature extraction part all happens here; all these sublayers consists of both excitatory and inhibitory neurons; (2) the learning layer, which consists of only excitatory neurons; (3) the classification later, which accumulates all the outputs from the learning layer, the whole system is illustrated in Figure 1. The whole system is illustrated in Figure 2. Note that the demo system has been reported for several conference papers such as [22], so we only briefly review the structure as follows in order to maintain the completeness for the section. 

### 4.1. Preprocessing

 The preprocessing process of the input images is divided into three steps: (1) face detection, eyes, and mouth location, (2) masking, and (3) illumination normalization. The first two steps are to provide normalized face region for further processing, and to remove irrelevant information such as the background and the hair, as well as some unnecessary accessories of a subject. Illumination normalization is essential, though human visual system can handle affective sensation in the extremely complex environment such as illumination variations almost effortlessly, illumination invariant processing, in general, is generally much more difficult than the first two steps. We assume the illumination effect ^2^ is processed along the two pathways separately, one way is to follow the main ventral route [30], where illumination effects will be discounted on the retina, usually viewed as preprocessing part, so as to facilitate the further processing, the other way is the bypass route where illumination and shadow information are passed from the retina directly to the IT area, where it helps to percept the 3D information of the scene ^3^. In our framework, only the main route preprocessing is considered. The illumination problem will be discussed and solved in the experiment section. 

### 4.2. From Retina to V1

 The neurons in first layer represent the On and Off cells of retina, act as edge detector, aimed at enhancing the high-contrast parts of a given image (high-pass filter), and usually can be implemented using two-dimensional difference of Gaussians (DoG), where frequency scales are chosen varying the standard deviation *σ* of the Gaussian curve:

(3) $$\begin{array}{c} \nabla^{2}G\left(x,y\right)=g\left(\frac{x^{2}+y^{2}-\sigma^{2}}{\sigma^{4}}\right)e^{-(x^{2}+y^{2}/2\sigma^{2})}. \end{array}$$

The neurons in second layer simulate the receptive fields (RFs) of V1 simple cells and complex cells, which can be interpreted as Gabor wavelet functions. In particular, the layer is composed of eight orientation maps for each frequency scale, each one being selective to different directions ($\overset{-}{0^{{}^{\circ}}}$, $\overset{-}{45^{{}^{\circ}}}$, 90°, 135°, 180°, $\overset{-}{225^{{}^{\circ}}}$, 27°, 315°) [17]:

(4) $$\begin{array}{c} \psi_{\mu ,\nu }\left(z\right)=\frac{{||k\left(\mu ,\nu \right)||}^{2}}{\sigma^{2}}e^{-{||k_{\mu ,\nu }||}^{2}{||z||}^{2}/2\sigma^{2}}\left[e^{ik_{\mu ,\nu }z}-e^{-\sigma^{2}/2}\right], \end{array}$$

where *μ*, *ν* define the orientation and scale of the Gabor kernels, *z* = (*x*, *y*), and we have *k*_*μ*,*ν*_ = *k*_*v*_ *e* ^*j*^ *ψ*_*u*_, where *k*_*v*_ = *k*_max⁡_/*f*′′ and *ψ*_*u*_ = *πu*/8. *f* is the spacing factor between kernels in the frequency domain.

### 4.3. From V1 to IT

 Learning dynamics happens at higher levels, the high dimensionality of the Gabor features makes dimension reduction techniques (such as PCA) needed to acquire a more sparse feature subset. Recent theoretical studies have shown that receptive fields that resemble cells in V1 can be learned (through biological optimization techniques) based on several sophisticated learning principles, for example, efficiency and sparseness [22, 24, 26] (minimizing the number of units active for any input), statistical independence [31]. ICA could be viewed as a reasonable option for capturing these statistics to form efficient representations of natural images, and research have shown that ICA could help to explain contextual phenomena in cortical neurons, such as response suppression, contrast gain control, and tuning property changes. In our framework, topographic ICA ^4^ is applied to mimicking such learning mechanism, for the merits of representing complex neuron behavior and explain the topography of the complex cells [25, 32].

The scheme of TICA model is illustrated in the framework of the overall system (see Figure 2), which can be viewed as an generative model with two levels. The classic ICA model is employed in the first level as the feature extractor for simple cells in V1, and in the second level (complex cells), a 2D topographic structure is defined to describe the correlations among the components in a small neighborhood. This can be accomplished by a neighborhood function *h*(*i*, *j*), which expresses the proximity between the *i*th and *j*th components. A simple illustrative example can be defined as

(5) h(i,j)={1,(|d(i)−d(j)|≤m)0,others.

The constant *m* defines here the width of the neighborhood, The neighborhood of the component with index *i* consists of those components whose indices are in the range *i* − *m*,…, *i* + *m*. If the distance between neuron *i* and *j* is less than a predefined constant *m*, then these two neurons are defined as neighbors and thus are nonlinear correlated. The neighborhood function *h*(*i*, *j*) is thus a matrix of hyperparameters. In this paper, we consider it to be known and fixed. Set *G*_*j*_(*I*) as the value of a small neighbor *j*, we have

(6) $$\begin{array}{c} G_{j}\left(I\right)=\sum_{i=1}^{n}h\left(i,j\right){\left(w_{i}^{T}I\right)}^{2}, \end{array}$$

where *I* is the image and *w*_*i*_ is the inverse of mixture coefficients matrix.

Using the ML(maximize likelihood) method, we can obtain the likelihood function as

(7) $$\begin{array}{c} L\left(W\right)=E\left[\sum_{j=1}^{J}\left(\sum_{i=1}^{n}h\left(i,j\right){\left(w_{i}^{t}I\right)}^{2}\right)\right]+T\text{lg}\left|W\right|. \end{array}$$

Notice that ∑_*i*=1_^*n*^ *h*(*i*, *j*)(*w*_*i*_^*t*^ *I*)^2^ could be considered as the energy of a neighborhood, possibly related to the output of a higher-order neuron as in visual complex cell models.

A simple gradient algorithm can be derived for performing the maximization of the approximation of likelihood function. The weight vector *w*_*i*_ is updated as

(8) $$\begin{array}{c} \Delta w_{i}\propto Ez\left(w_{i}^{T}z\right)r_{i}, \end{array}$$

where *z* = *Vx* = *VAS* is the data prewhitening process. *V* = *E*(*xx* ^*T*^)^−1/2^ is the whitening matrix. And we have

(9) $$\begin{array}{c} r_{i}=\sum_{k=1}^{n}h\left(i,k\right)p\left(\sum_{j=1}^{n}{\left(w_{j}^{T}z\right)}^{2}\right). \end{array}$$

The function *p* is the derivative of *P*, here, we define a exponential distribution $P(y)=\alpha \sqrt{Y}+\beta$, where *α* is the scaling constant while *β* defines the normalization. The orthogonalization and normalization of the weight matrix *W* can be accomplished by letting

(10) $$\begin{array}{c} W⟵{\left(WW^{T}\right)}^{-1/2}W. \end{array}$$

Finally, after the learning is over, the original mixing matrix *A* can be computed by inverting the whitening process as

(11) $$\begin{array}{c} A={\left(WV\right)}^{-1}=V^{-1}W^{-1}. \end{array}$$

For details, please refer to [25]. 

### 4.4. Learning and Neural Computation Output

 Supervised learning follows in procedure, the model tries to update the weight connection with output neuron map by modifying the mean value of the synaptic weight between the neuron excited *i* and the preselected neuron *j*, thus, Δ*w*_*j*,*i*_ = mod⁡^order(*a*_*j*_)^/*N*. Note that the neurons in the output maps share the same synaptic weights. The result is the neurons in the output map will respond to the average pattern of the training samples, taking the metric of being robust to the spatial position of the detected face or facial expression and computationally convenient, while being insensitive to the case in which the patterns are known to have high variance, such as recognizing facial expression of the subject with variant scale and illumination in the complex scene.

Last layer creates the number of neuronal maps corresponding to the number of pattern class presented to the network. Neurons are trained to respond selectively to the presence of a given input (face, facial expression, etc.) at the center of their receptive field. Following the lateral inhibition theory, whenever a neuron for a predefined category spiked, all the neurons of the other neuronal maps (standing for other categories) in a zone centered on the neuron's location will receive inhibitory pulses (fitting Gaussian curve theoretically), forming a discriminative classifier. 

## 5. Empirical Evaluation

 In this section, we evaluate our framework on several datasets, for the aim of fair evaluation and overall performance, we try to evaluate the approach from different aspects, such as frontal facial expression recognition, and facial expression recognition under the constraints of illumination variation. In an effort to make a comparison, we also provide several other bench systems and test evaluation methods. 

### 5.1. JAFFE Database Experimental Results

 The first experiment is the test on the Japanese female facial expression (JAFFE) dataset [33]. The JAFFE dataset contains 213 images of seven facial expressions which include six basic facial expressions and one neutral expression posed by ten Japanese models. JAFFE is used as the benchmark database for several methods. Also, for its pure Japanese characteristic, sometimes it is also used for the comparison research for cross-culture exploration such as Dailey et al's work in [20, 21]. JAFFE also stands out for the psychological view that woman tend to percept and display more explicit emotional facial expressions than man. Therefore, it is reasonable to begin the experiment evaluation from this database.

Table 1 summarizes the performance of the proposed method and other published result of benchmark systems^5^, and note that our results are superior to other methods [34, 35], yielding satisfactory results. 

### 5.2. CUN Frontal Facial Information Database Experimental Results

 The second experiment presented here is the evaluation of the approach on a newly created face database that the Minzu university of China has designed and constructed, namely, a large-scale racially diverse face database, the CUN face database ^6^, which covers different source of variations, especially in race, facial expression, illumination, backgrounds, pose, accessory, and so forth Currently, it contains 112,000 images of 1120 individuals (560 males and 560 females) from 56 Chinese “nationalities” or ethnic groups. The aims of the dataset are listed as follows [36].

**Figure 3:**
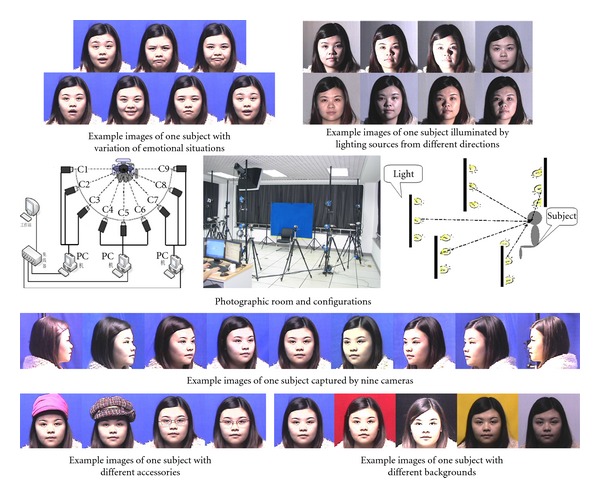
Diagram showing the whole configuration of the CUN face database. To capture face images with varying poses, expressions, accessories, and lighting conditions, a special photographic room with the dimension of 8.0 m length, 8.0 m width and 3.5 m height is set in our laboratory, and the necessary apparatuses are configured in the room including a multicamera system, a lighting system and control device, accessories, and various backgrounds. Note that in an effort to simulate the ambient illumination, two photographic sunlamps of high power covered with a ground glass are used to irradiate to the rough white ceiling, which can obtain more uniform lighting and mimic the normal outdoor-lighting environment (overhead lighting sources). To generate various directional lighting conditions needed, we set up a lighting system of 15 fluorescent lamps in the photographic room using multiple lamps and lamps hades, in a semicircle configuration [22].

1. To provide the worldwide scholars of face recognition with exhaustive ground-truth information in a cross-race face database. While most of the current database mainly consists of Caucasian people, we mainly focus on the“cross-race effect” during the experiment.
2. To understand culture specific difference in facial expression production and interpretation, which have been long viewed as a crucial interlink between individual and social communication.
3. To provide facial data for a brain-computer interface (BCI) system project, in which the goal is to collect EEG and facial expression, either voluntary or controlled, of the subjects excited by the selected audio-visual stimulus, recorded with a scalp EEG device, and to analyze and determining nonlinear-correlation between aroused emotion and its manifestation on facial expression.  Figure 3 shows the configuration of the photographic room, including lamps, camera system, and so forth, and some typical images of subjects.

**Figure 4:**
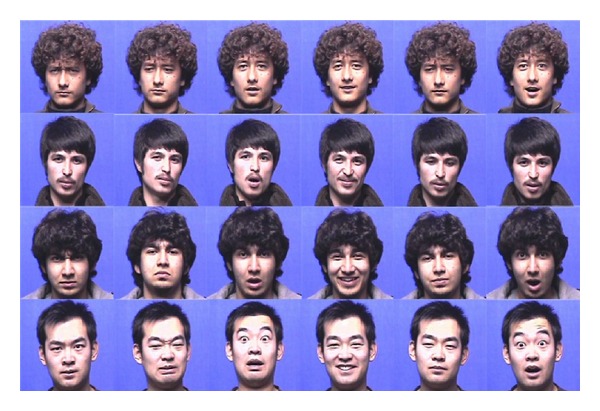
Racially diverse facial expressions implying complicated neurodynamics and implicit cultural influence (raw photos) [22].

The first experiment we carried out was on a subset of seven frontal datasets (six typical facial expressions plus one neutral expression), on which some of the most commonly used baseline facial recognition algorithms and our proposed method were evaluated. 300 subjects, each of which contains more than 14 frontal facial expression images, were employed. Note that some subjects share similar facial expression appearances, but most of them have racially diverse and variant expression intensity (Figure 4). We used 70% (10/14) of images of each class for training and a varying fraction of the remaining images for testing. The some part of experimental results and introduction about the face databases have been appeared on the conference paper [22, 36] and our approach has achieved promising results comparable to the top performances of the state-of-the-art methods such as [31, 37, 38].

During the experiment, we found that for all the six facial expressions, the happy expression and the surprise expression are the easier expressions to be recognized whereas the fear expression is the most difficult expression to be recognized, which is consistent with the psychological results such as [39]. Another notable fact about the specific category is that the recognition rate for fear, disgust, and surprise information is relatively lower than some other western facial expression datasets such as Cohn-Kanade AU-Coded Facial Expression Database [40], on which we have some empirical experimental results. Once again, the situation could be accounted by the culture specific explanation that it is relatively easy to analyze for explicit or western stylized negative facial expressions. Also, some behavior and event-related potential experimental results [41, 42] support this conclusion for emotional face recognition confusion, that is, eastern asian people tend to have difficulty differentiae fear and disgust emotional expression, while western people do not have that problem. All together, the results indicate the useful potential of the proposed method for dealing with such kind of problem. However, our method still showed satisfactory results on average ^7^. 

### 5.3. Illumination Variation Facial Expression Recognition Experiment

 The second experiment we consider here for the CUN dataset was the evaluation of the proposed approach on the facial expression recognition under illumination variation. While the most current researches are restricted on the frontal view with normal illumination condition, facial expression recognition with variant illumination conditions is a challenging research topic which has recently started to attract the attention of the research community. However, few work on this issue have been done in the past several years because of its technical challenges and the lack of appropriate databases. We choose CUN illumination variation subset, 30 subjects with five pose angles (we also consider the shadow effects caused by pose variation), 3 illuminations and 6 facial expressions are selected randomly for the generic training and the rest are used for testing. We consider the following experiment procedures: (1) same illumination, same pose, which represents the traditional fixed scenario, and (2) different illumination, same pose, in which the subject's facial expression should be recognized with the same pose (say, frontal, thus means 0°) while the illumination varies from side to central). Experimental results are listed in the Table 2 (%, the decimal point is omitted).

From the table we can see that this task is indeed very challenging for both databases, if the pose and illumination conditions are both extreme, then almost none of the face would be visible, let alone the facial expressions. If the subject's pose is 0°, and illumination angle varies from 0° to 90°, then the problem turns into the simple frontal facial expression recognition under different illumination angle, and we obtain acceptable results^8^, even in the extreme illumination angle 90°, that is because face image is generally symmetrical, thus it is relatively easy to recognize, even for facial expressions (Usually this viewpoint holds, when a subject's emotional expression is not symmetrical across his face, it is possible that he/she is trying to pretending to hide the inner emotional status, or express it unnaturally.). But when subject's pose is more than 30°, the symmetry is destroyed, and being lack of structure information makes the algorithm hard to extract reliable information, at the same time, casted shadow effects and attached shadow will make the recognition worse, even when the degraded image is partially restored by normalization preprocessing algorithms^9^. During the experiment, our method performs satisfactory only when test image is relatively integrated, which means the shadow effect influences image not too much ^10^ and it also indicate that the so-called “immediate vision” (meaning fast categorization without eye movements or attention) has its limitations for dealing with illumination variant problem, which could be sent to the higher cognitive, attention demanding area for processing. However, it should also note that compared with the unnormalized image, the recognition result of the approach did not degrade much, indicating the robustness for the performance of the system (invariance to the illumination variation). 

## 6. Discussions, Summaries, and Future Directions

### 6.1. Summaries

 In this paper, we focus on a potential form of cortex like framework of fast categorical decision making for facial expression recognition. Our hypothesis is that rapid decision making is feed forward in V1, and neural encoding's inborn physiological behavior will reduce the redundant information, increase selectivity, while maintain invariance, thus, in a way that is consistent with perceptual performance, therefore, the system described in this work is based on a consensus among neuroscientists, psychologists and on fitting available experimental data. It falls into a family of feedforward models of object recognition that tries to duplicate the tuning properties of neurons in several visual cortical areas. The model consists of several levels, the type of function of each layer is summarized separately as follows.

The first layer mimics the biological properties of On and Off cells of retina, enhancing the high-contrast parts of a given image, using two-dimensional difference of Gaussians, performing the role of highpass filters.

The second layer consists of applying Gabor filters to the input from the first one, mimicking the processing by simple cells in the primary visual cortex. Olshausen and Field demonstrated that optimizing a simple sparse coding scheme over a set of natural images produces a set of edge filters similar to Gabor filters [26, 43]. Thus, the output of Gabor filters on the input images should have the desirable sparse property.

The third level does something unorthodox for traditional computer vision models, it tries to remove the redundant representations of information while preserving the maximization of mutual information, revealing the underlying independent components of the inputs, a typical efficient coding approach. Hence, generative statistical models such as TICA would be the obvious choice at this stage. The motivation is trigged by the small world connectivity (meaning sparsely distributed, locally stimulated computation phenomena founded in the cortex) and efficient coding hypothesis (meaning early visual processes should take advantage of the statistical regularities or redundancies of inputs to represent as much information as possible given limited neural resources) suggest that energy efficiency can be used to account for the sparse coding theory [1, 2, 26, 43]. It has been noticed that the fraction of ever, strongly active neurons is relatively small for the stimuli, the so-called sparse coding theory demonstrates that the neurons in primary visual cortex form a sparse representation of natural scenes in the viewpoint of statistics. Vinje and Gallant et al acclaimed that neurons in the early visual system should have the unique ability of decorrelating the incoming visual signals, removing the redundant information, in an effort to maximize the information transmission [27]. Although it is still not clear how to model the entire visual cortical system to selectively amplify important features to facilitate discrimination, it has been widely accepted that sparse-coding-based neuron system improves neural information processing and cortex perception.

The last stage of our system is a standard information accumulation and decision part following the original SpikeNet model, corresponding to V4-IT, neurons are trained to be selective to predefined categories (one neuronal map for each individual). 

### 6.2. Discussion

Ongoing efforts within cognitive neuroscience, pattern recognition, and advanced human-machine system have been directed toward the building of computationally intelligent models using simulated neuron units as basic building blocks. Such efforts, inspired by the standard design of cortex-like mechanism and traditional artificial neural networks, are limited by the difficulties arising from single functional performance and massive computational inconvenience, especially when dealing with large-scale, complex-pattern recognition problem. Our proposed model, on the other hand, suggests that, by combining the models and tasks of cognition with modern neurocomputational approaches, the neurosystematic approach to the study of cortex-like mechanism has the potential to overcome the aforementioned difficulties, to extend our knowledge of brain mechanisms underlying the cognition analysis, and to advance theoretical models of how we recognize face or, for example, perceive other people's emotion in a rich, dynamic, and complex environment, providing a new starting point for improved models of visual cortex-like mechanism, informed by the formal mathematical approach of neuron models and constrained by known visual ventral pathway models. Researches have already begun to illustrate how this combination can act directly on several specific application tasks.

### 6.3. Future Directions

 There seem to be at least four directions that could be followed to further improve the performance of the cortex-like mechanism here.

First, as future direction, in an effort to to improve the use of biologically plausible realistic neural networks for pattern analysis, adaptation is highly required. It has been experimentally shown that V1 receptive fields adaptively change with the input stimuli so as to increase the information carried by the neural response about the filtered stimulus [44], which means neural encoding is adaptive, and this adaptive filtering process actually affects the spatial frequency composition of the neural filter, thus enhances information transmission in visual cortex, in agreement with optimal neural encoding. It would be convenient if this adaption filtering mechanism could be integrated into the system, since current models lack of adaption, one way would be using adaptive gabor filters, or applying local adaptive, global stable kernel methods.

Second, very recent findings from neuroscience by Tsao, Freiwald et al. suggest that successive stages in the face network may perform a stepwise transformation: from selectivity for viewpoint, regardless of identity, to selectivity for identity, regardless of viewpoint [45–47]. The general implication is that earlier processing stages in the ventral pathway carry information about generic categories (e.g., face versus nonface, a typical fast categorical decision-making task, also in accordance with the current theoretical models and experimental founding) and viewpoint, whereas later processing stages carry information about individual exemplars (e.g., Roger versus Michael), eliminating viewpoint information to achieve invariant recognition, suggesting that invariant scale and position feature descriptors (for example, operators like SIFT) may be necessary to be introduced to form additional layer. The same concept would undoubtedly be suitable for the robust facial expression recognition.

Third, the current spiking neuron models are deterministic, restricting them from describing and modelling large-scale, dynamic, and stochastic process, while as spiking process in biological neurons are stochastic by nature (neurons spike or not, synapses connected or not, transmission channels open or not, etc), it would be appropriate to look for new inspirations to enhance the current SNN models with probabilistic parameters, forming probabilistic spiking neural networks (pSNNs). For example, adding probabilistic parameters to the spiking neuron model (such as Izhivich's SNN model) will mimic the behavior of the cortical neurons in vivo, in which the parameters are used to control synapses established during spiking generation and transmitting. And the Hebbian learning rule can be employed for controlling the probabilistic parameters self-adaptation and connection weights associated with the synapses which are established using Thorpe's rule during the network learning procedure. Such a pSNN model will exhibit more explicit behavior and robust performance than the original model and deterministic network organizations. Some experimental results have already been presented for its efficiency for performing functions difficult to be implemented using conventional models [48].

Finally, another promising direction would be the modification of SNN architecture. All the existing neuromodels will emphasis on multilayer feedforward transformed, hierarchical layout structures which is based on the traditional physiological experimental founding. However, very recent experiment has found that processing within visual feature patches and additional recurrent processing between patches at different levels of the processing hierarchy (parallel) are likely further mechanisms that may bring about more elaborate representations [46]. It would be important and necessary to use recurrent spiking neural network to describe the dynamics of the process. However, this means that we need to discard the original architecture because spike propagation adopted in those straight forward models such as SpikeNet is feedforward only and iterative processes cannot occur in the sense that, even if lateral interactions are present in the last processing stage, each neuron can only fire once. In order to solve the more complicated, dynamic facial information analysis problem, the fundamental redesign of the neuron models [49] is by all means important and necessary. 

## 7. Conclusions

 Building a intelligent human-machine system has always been a dream for scholars for centuries, there has been a great deal of interest in studying the emulation of brain-like process for the purpose of pattern recognition. In this paper, a practical implementation is presented using a highly structured cortex-simulated system, which can be simply described as feedforward, hierarchical simulation of ventral stream of visual cortex using biologically plausible, computationally convenient spiking neural network system. The proposed cortical-like feedforward hierarchy framework has the merit of capable of dealing with complicate pattern recognition problems. Discriminative visual features are grouped and refined along stepwise levels. The independent component analysis can perform better than other descriptors for facial expression recognition, since the efficient coding approach-based representation is localized and sparse, providing highly discriminative and efficient feature descriptors. We demonstrate our system on several facial expression recognition tasks. Of note, small structure modifications and different learning schemes allow for implementing more complicated decision system, showing great potential for discovering implicit pattern of interest and further analysis. 

## Figures and Tables

**Table 1:** Classification results for the JAFFE dataset with our method and comparison with other methods.

Feature Extraction Methods	Recognition Rate (%)
PCA + SVM	93.43
ICA + SVM	93.35
LDA + SVM	91.27
2D-LDA + SVM	94.13
Ours	97.35

**Table 2:** Recognition accuracy of illumination variant facial expression dataset results (with and without normalization).

Name			CUN facial	Expression subset			
		Un-normalized			Normalized		
		Illumination Angle			Illumination Angle		Ave
Degree	0°	45°	90°	0°	45°	90°	
Pose	H/Sa/Su/D/F/A	H/Sa/Su/D/F/A	H/Sa/Su/D/F/A	H/Sa/Su/D/F/A	H/Sa/Su/D/F/A	H/Sa/Su/D/F/A	(%)

0°	85/76/79/67/65/82	57/53/55/55/48/63	66/57/58/62/51/59	85/76/79/67/65/82	66/52/65/59/47/66	67/55/61/56/47/62	63/64
30°	67/65/61/53/42/57	53/51/50/51/43/55	51/55/58/55/50/48	66/59/60/72/48/62	60/65/59/52/45/56	46/45/40/45/32/42	53/53
45°	63/56/52/55/35/51	45/55/47/45/43/50	44/42/42/45/43/40	66/43/50/51/52/55	58/53/57/51/42/55	48/44/39/40/38/43	47/49
60°	48/46/44/47/38/45	42/45/45/45/32/41	36/36/33/35/35/39	57/52/45/49/59/61	50/45/44/49/40/51	41/40/36/35/22/32	40/45
90°	33/33/33/33/17/33	33/37/31/35/25/33	37/30/31/25/18/32	38/39/37/33/40/42	36/33/40/33/38/33	35/37/35/35/31/33	31/36

Average (%)	59/55/54/51/40/54	46/48/46/46/38/48	47/44/45/44/39/44	62/54/54/54/53/60	54/50/53/49/42/52	47/44/42/42/34/42	47/49

Notes	H-Happiness	Sa-Sadness	Su-Surprise	D-Disgust	F-Fear	A-Anger	

## Endnotes

1. Note that some models [9, 10, 24] have proposed implicit or explicit processing in early visual areas to create a SIFT like image representation, although the need for such explicit preprocessing has been questioned on the basis of neurophysiological evidence. However, very recent experimental results support this viewpoint [47].
2. Generally, illumination and shadows are classified into two types: cast shadows and attached shadows. Cast shadows arise when a light source is obstructed by a part of the same or another object, such as shadows casted by the nose which is caused by the side light source, and they could yield global constraints on surface depths. On the other hand, attached shadows arise when “the angle between a surface and a light source direction is obtuse, and they yield local constraints on surface [50].” For details, see to [50].
3. Several models and neurophysiological experiments on macaque monkey inferotemporal cortex have shown that explicit structural coding mechanism for three-dimensional object shape can be embodied by multiple object surface fragments, view-dependent theories and spatial relationships between shape elements inferred by the illumination-dependent theories [47, 51].
4. Recent physiological founding [25] shows that independence does not hold by simply applying the ICA model to the image due to the existence of strongly nonlinear correlations among the extracted components and topographic organization structure among the neighbor neurons.
5. Similar experimental results have been published previously in the conference paper in [22], here we rearranged the whole experiment and acquired slightly different results. Notice that experimental results are benchmarks cited from [34].
6. The former name of the Minzu University of China is called central university of nationalities, Minzu is the Chinese pronunciation of ethnic groups or nationalities.
7. Experimental procedures may vary from one to another (e.g., data used, cross-validation, leave-one-out, preprocessing, scale normalization, etc.). Therefore, comparisons should be taken cautiously.
8. Lower results than the original paper in [22] due to the different subjects and preprocessing methods, during the 0° situation, the direction light at 0° position is open, overlapped with the two photographic sunlamps (see Figure 3 for illustration), causing light spots which blurred some essential features.
9. For the illumination normalization problem, we applied a human perception-based image processing method which could provide color constancy and dynamic range compression simultaneously in the preprocessing section, specifically, multiscale retinex approach, or MSR algorithm [52], is applied and written as follows:
  (12) Fi(x,y)=∑n=1NWn︸MSR×log⁡⁡[Si(x,y)]−log⁡⁡[Si(x,y)∗Mn(x,y)]︸SSR,
  where the expression within the summation represents an single-scale retinex model. *S*_*i*_(*x*, *y*) is the image distribution in *i*th color spectral band. *N* stands for the scale numbers and *i* represents the three color bands. *W*_*n*_ are the weighting factors for the scales. *M*_*n*_(*x*, *y*) are the surround Gaussian function defined by
  (13) $$\begin{array}{c} M_{n}\left(x,y\right)=K_{n}\exp\left[\frac{-\left(x^{2}+y^{2}\right)}{\sigma_{n}^{2}}\right], \end{array}$$
  where *σ*_*n*_ determines the space and amplitude of the function. *K*_*n*_ is applied to make sure ∫∫*F*(*x*, *y*)*dx* *dy* = 1. Experimental results show that it produces a much better image in both term of color and dynamic range than the other similar methods such as histogram equalization (HE) and logarithmic method.
10. During the capture of the image, if the illumination variation is controlled by flashlight (like some other face database such as CMU PIE [53]), then these strict point light source could be relatively easier to be erased by some preprocessing algorithm such as simple white balancing or MSR algorithm, whereas in CUN face database, the direction light system (fifteen fluorescent lamps controlled by a switch matrix) is not point light source actually, making it hard to discount the effects, even by MSR algorithm.
